# A novel Foxp3-related immune prognostic signature for glioblastoma multiforme based on immunogenomic profiling

**DOI:** 10.18632/aging.202282

**Published:** 2021-01-10

**Authors:** Xiao-Yu Guo, Guan-Hua Zhang, Zhen-Ning Wang, Hao Duan, Tian Xie, Lun Liang, Rui Cui, Hong-Rong Hu, Yi Wu, Jia-jun Dong, Zhen-Qiang He, Yong-Gao Mou

**Affiliations:** 1 Department of Neurosurgery/Neuro-oncology, Sun Yat-sen University Cancer Center, State Key Laboratory of Oncology in South China, Collaborative Innovation Center for Cancer Medicine, Guangzhou 510000, China; 2 Department of Cerebrovascular Surgery, The Third Affiliated Hospital, Sun Yat-sen University, Guangzhou 510000, China; 3 Department of Neurosurgery, Jiangmen Central Hospital, Jiangmen 529030, China

**Keywords:** glioblastoma multiforme, Foxp3, regulatory T cells, immune prognostic signature, nomogram

## Abstract

Foxp3^+^ regulatory T cells (Treg) play an important part in the glioma immunosuppressive microenvironment. This study analyzed the effect of Foxsp3 on the immune microenvironment and constructed a Foxp3-related immune prognostic signature (IPS)for predicting prognosis in glioblastoma multiforme (GBM). Immunohistochemistry (IHC) staining for Foxp3 was performed in 72 high-grade glioma specimens. RNA-seq data from 152 GBM samples were obtained from The Cancer Genome Atlas database (TCGA) and divided into two groups, Foxp3 High (Foxp3_H) and Foxp3 Low (Foxp3_L), based on Foxp3 expression. We systematically analyzed the influence of Foxp3 on the immune microenvironment. Least Absolute Shrinkage and Selection Operator (LASSO) Cox analysis was conducted for immune-related genes that were differentially expressed between Foxp3_H and Foxp3_L GBM patients. We found a differential expression of Foxp3 in high-grade glioma tissues. The presence of Foxp3 was significantly associated with poor OS. From the four-gene IPS developed, GBM patients were stratified into low-risk and high-risk groups in both the training set and validation sets. Furthermore, we developed a novel nomogram to evaluate the overall survival in GBM patients. This study offers innovative insights into the GBM immune microenvironment and these findings contribute to individualized treatment and improvement in the prognosis for GBM patients.

## INTRODUCTION

Gliomas are the most common primary tumors in the human central nervous system (CNS) with more than half of them being World Health Organization (WHO) grade IV glioblastomas multiforme (GBM) [1]. Because glioblastomas are highly heterogeneous and invasive, surgery alone cannot eradicate the tumor and therefore, subsequent radiotherapy and chemotherapy are still required [2]. Although temozolomide has been called the biggest advancement in GBM chemotherapy [3], its therapeutic effect is still not ideal. The median survival time of GBM patients is only 14 months after standardized treatment [4].

The application of immunotherapy in non-small cell lung cancer and melanoma has provided a potential new approach to treating GBM [5, 6]. Studies of the anatomical structure of the CNS endolymphatic duct have shown that the CNS is not an immune exempt area [7]. CNS tumors can also be infiltrated by peripheral lymphocytes, which can have a meaningful therapeutic effect on existing CNS tumors. Nonetheless, GBM immunosuppressive microenvironment limits treatment effect. The glioblastoma tumor uses a variety of immunosuppressive mechanisms to promote growth and progression. GBM cells can downregulate immune activity by secreting immunosuppressive factors such as IL-1, TGF-β, and CSF-1 [8, 9]. They can also limit the compromise the immunity by expressing CD95, CD70, PD-L1, and other immunosuppressive factors [10, 11]. A number of clinical trials against these targets have been applied to glioblastoma patients, but the therapeutic effect is not satisfactory.

Foxp3^+^ regulatory T cells (Treg) play an important role in the glioma immunosuppressive microenvironment. Foxp3^+^ Tregs of glioma can bind to CD80 or CD86 via CTLA-4 to suppress T lymphocyte activity [12]. Foxp3 can also induce HO-1 expression resulting in the suppression of T lymphocyte proliferation [13]. In gliomas, Treg can inhibit dendritic cells, antigen-presenting cells, and other lymphocytes by inhibiting the secretion of IL-2, and IFN-γ, and promoting the secretion of TGF-β, IDO thus, creating an immunosuppressive microenvironment [14]. Although some studies have explored the immunosuppressive effect of Treg in glioma, the relationship between Foxp3 expression and the immune response has not yet been discussed in GBM.

In this study, we first performed an immunohistochemical examination for Foxp3 of tissue microarray slides of 72 high-grade glioma patients and analyzed the patients’ survival time. We then obtained RNA-seq data from The Cancer Genome Atlas (TCGA) and divided them into two groups based on Foxp3 expression, Foxp3 High (Foxp3_H), and Foxp3 Low (Foxp3_L). The enrichment levels of the 29-immune signature (Supplementary Table 2) which represented diverse immune cell types, functions, and pathways in each GBM sample of the two groups were then quantified [15]. The gene expression profiles were also analyzed to identify differentially expressed immune genes (DEIGs) between Foxp3_H and Foxp3_L. Subsequently, we used the Least Absolute Shrinkage and Selection Operator (LASSO) Cox regression analysis to construct an immune prognostic signature (IPS). Finally, a robust predictive nomogram model was developed to estimate overall survival (OS) for patients with GBM.

## RESULTS

### Foxp3 expression is associated with prognosis

**Figure 1:**
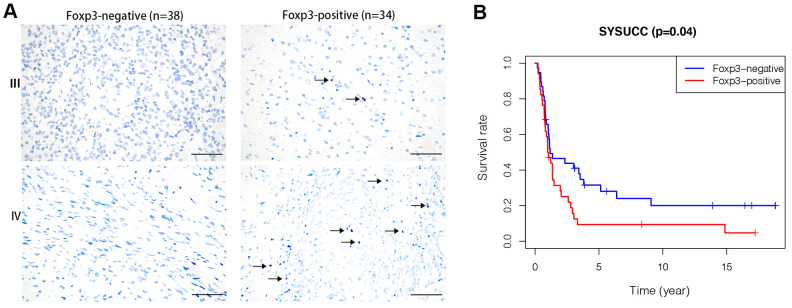
**Foxp3 expression in high-grade gliomas from SYSUCC and survival analysis.** (**A**) Foxp3 expression in 72 high-grade gliomas from SYSUCC, black arrows shows the positive cells. The scale bar represents 50 μm. (**B**) Comparison of survival prognosis between 34 Foxp3-positive patients and 38 Foxp3-negative patients from SYSUCC (Log-Rank test).

Clinicopathological details of patients in the prognosis cohort and their association with Foxp3 expression are summarized in Supplementary Table 1. IHC staining for Foxp3 was positive in 34 (47.2%) patients and negative in 38 (52.8%) patients. Foxp3 expression was very different in high-grade glioma tissues (Figure 1A). In the survival analysis, Foxp3 positivity was significantly associated with poor OS (*p*=0.04) (Figure 1B).

### Immunogenomic analyses between Foxp3_H and Foxp3_L

**Figure 2:**
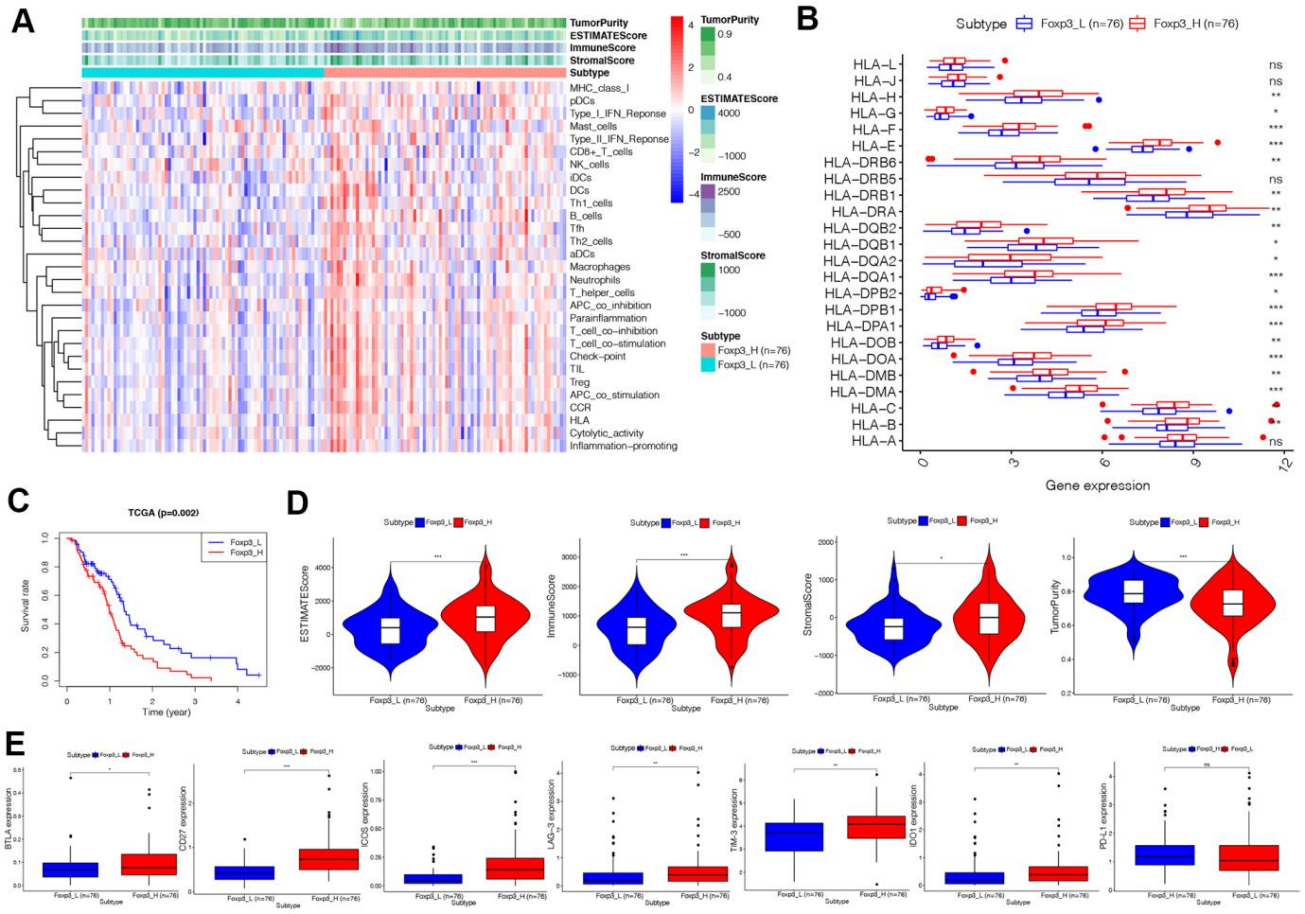
**Immunogenomic analyses of Foxp3_H (n=76) and Foxp3_L (n=76) from TCGA.** (**A**)The enrichment levels of the 29-immune signatures by ssGSEA score in each GBM sample. ESTIMATE was used to evaluate tumor purity, stromal score, and immune score. (**B**) Comparison of the expression levels of HLA genes between Foxp3_H and Foxp3_L using ANOVA test. (**C**) Comparison of survival prognosis between Foxp3_H and Foxp3_L from TCGA using the Log-Rank test. (**D**) Comparison of the Immune score, Stromal score, ESTIMATE score, Tumor purity between Foxp3_H, and Foxp3_L using Kruskal–Wallis test. (**E**) Comparison of immune checkpoint gene expression levels between Foxp3_H and Foxp3_L using ANOVA test. *P < 0.05, **P < 0.01, ***P < 0.001.

The 152 patients in this study were equally divided into two groups: Foxp3_H and Foxp3_L, of 76 patients each. The ssGSEA score [16, 17] was applied to examine 29 sets of immune-associated genes (Supplementary Table 2) [15] representing different immune cell types, functions, and pathways in each GBM sample of the dataset. From the ssGSEA results, we found that immune cells, functions, and pathways were enriched in Foxp3_H (Figure 2A).

We then analyzed the expression of HLA genes and immune checkpoint genes. It was found that the expression of HLA genes was higher in Foxp3_H than Foxp3_L, except HLA-L, HLA-J, HLA-DRB5, and HLA-A (ANOVA test, *P*<0.05) (Figure 2B). Most immune checkpoint genes expressed were significantly higher in the Foxp3_H group, except PD-L1 (ANOVA test, *P*<0.05) (Figure 2E). These results indicated that patients in Foxp3_H suffered from an immunocompromising condition. This implied that the Foxp3_L might better respond to immune checkpoint inhibitors than the Foxp3_H.

Survival analyses showed that clinical outcomes were distinct between Foxp3_H and Foxp3_L (Figure 2C). The Foxp3_L had a better survival prognosis than the Foxp3_H (Log-Rank test, *P*=0.002). Additionally, we compared the immune score, stromal score, ESTIMATE score, and the tumor purity between Foxp3_H and Foxp3_L. We found that the immune scores (Kruskal–Wallis test, *P* < 0.001), ESTIMATE scores (Kruskal–Wallis test, P < 0.001), and stromal scores (Kruskal–Wallis test, *P* < 0.05) were higher in the Foxp3_H group whereas the tumor purity was higher in the Foxp3_L group (Kruskal–Wallis test, *P* < 0.001) (Figure 2D). Overall, these results indicated that Foxp3_H had more immune and stromal cells, while Foxp3_L had more tumor cells.

### Identification of differentially expressed immune genes

Data were analyzed using Rstudio software (Version 1.2.5001) to confirm the DEGs between Foxp3_H and Foxp3_L. A total of 294 genes were identified by the criteria of log2 |fold change| ≥1 and FDR <0.05; among them, 261 genes were upregulated and the other 33 genes were downregulated (Supplementary Figure 1).

**Figure 3:**
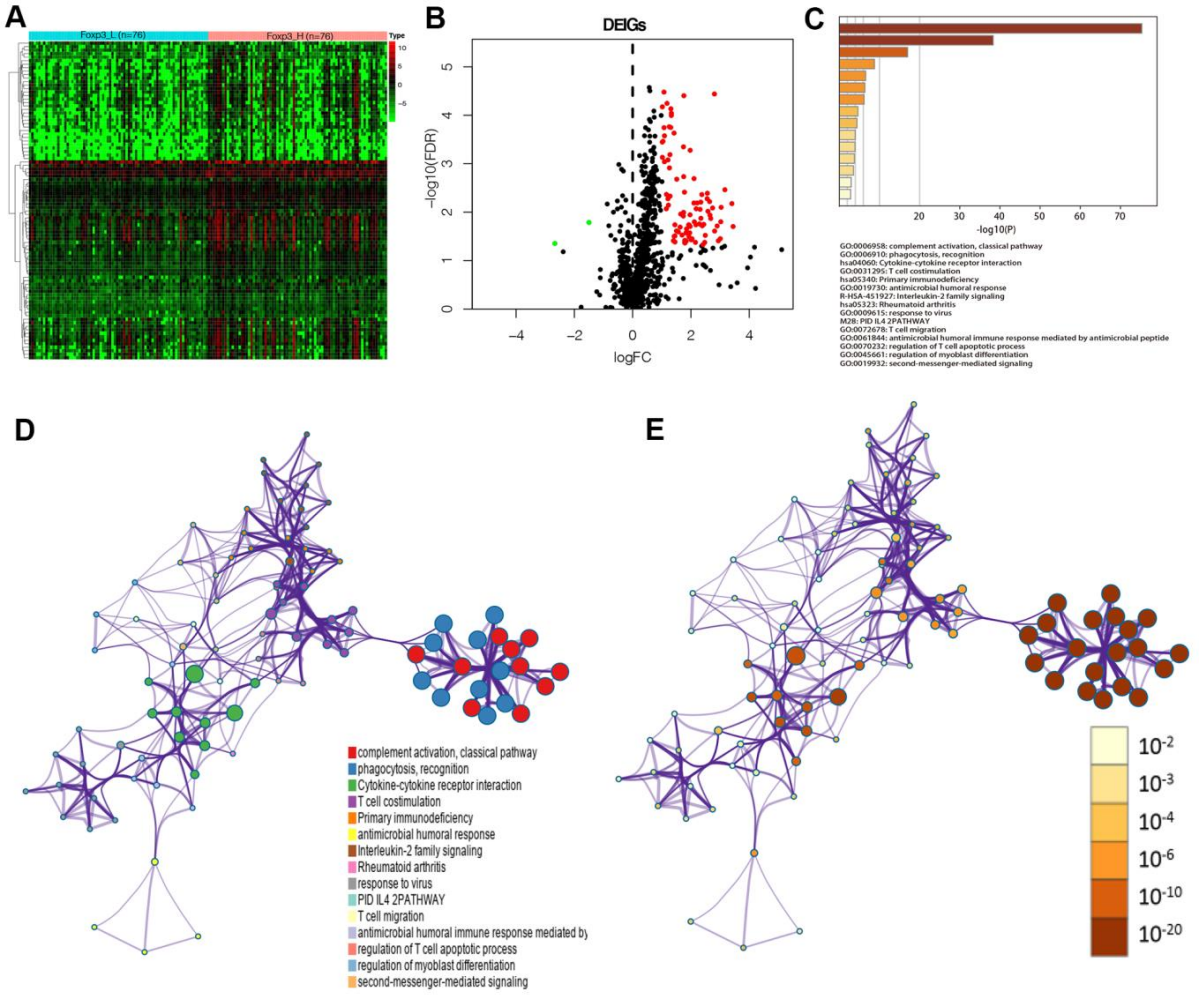
**Identification of Foxp3-associated differentially expressed immune genes.** (**A**) Heatmap of immune genes differentially expressed between Foxp3_H (n=76) and Foxp3_L (n=76). (**B**) Volcano plot of 91 immune genes differentially expressed between Foxp3_H and Foxp3_L. (**C**) Heatmap of enriched terms across input gene lists, colored by P-values. (**D**) Enriched terms are colored by cluster ID, where nodes that share the same cluster ID are typically close to each other in DEIGs. (**E**) Enriched terms are colored by P-value, where terms containing more genes have a more significant P-value in DEIGs.

Ninety-one differentially expressed immune genes (DEIGs) were then selected using the ImmPort database [18] (Figure 3A, 3B). The DEIGs were uploaded to the Metascape website to identify Gene Ontology (GO) Terms and Kyoto Encyclopedia of Genes and Genomes (KEGG) pathways. The terms were considered significant and grouped into clusters when *P* <0.01 and the numbers of enriched genes ≥3, respectively based on their membership similarities. The terms with the best *p*-values were chosen from each of the 20 clusters, with not more than 15 terms per cluster and 250 terms in total. The DEIGs were mainly enriched in GO:0006958: complement activation, classical pathway, GO:0006910: phagocytosis, recognition, GO:0031295: T cell co-stimulation (GO terms), and hsa04060: cytokine-cytokine receptor interaction (KEGG) (Figure 3C). Each node represented an enriched term and was first colored by its cluster ID (Figure 3D) and then by its *P*-value (Figure 3E).

### Construction of the Foxp3-related immune prognostic signature

**Figure 4:**
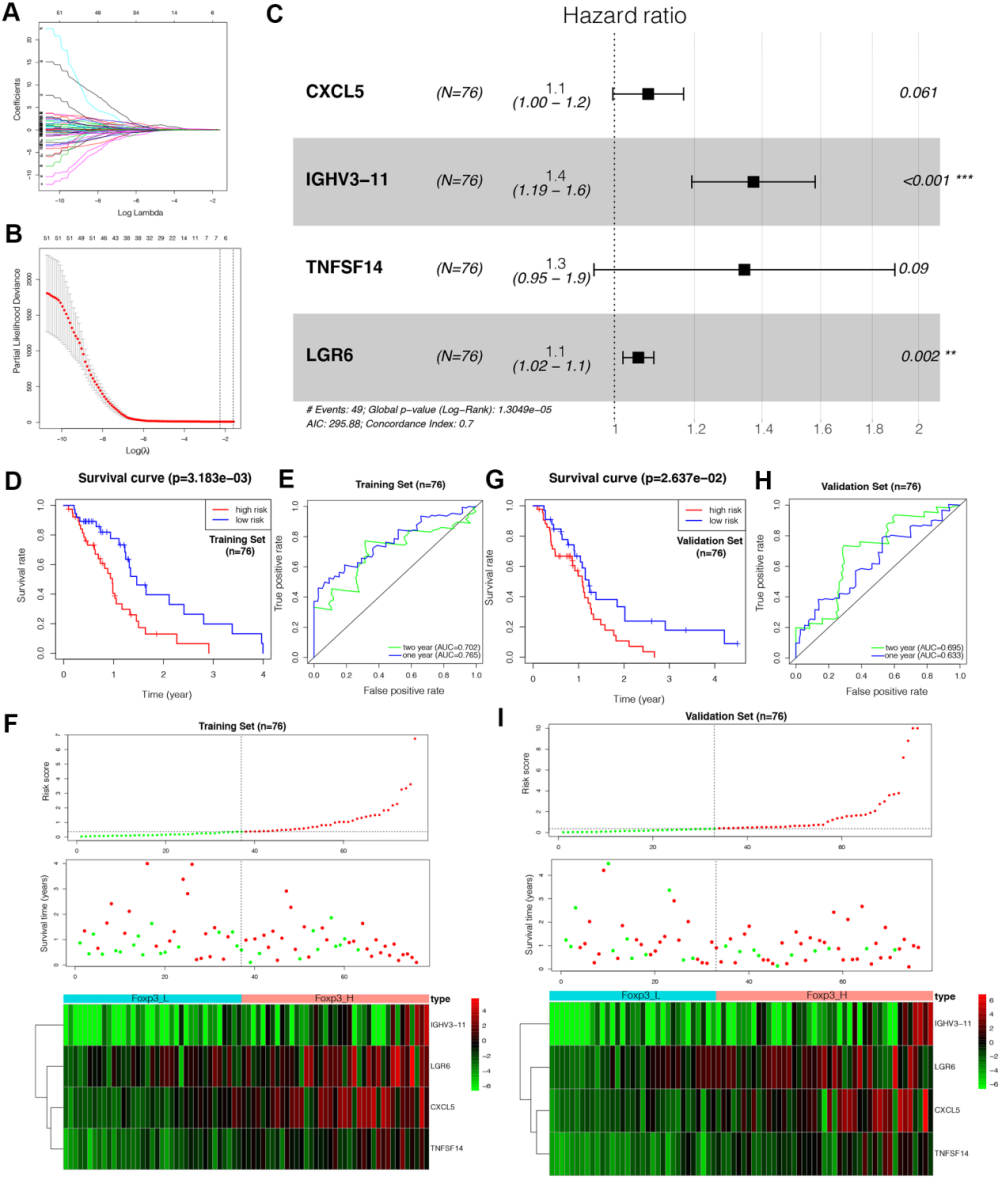
**Construction of the Foxp3-related immune prognostic signature (IPS).** (**A**–**C**) LASSO Cox analysis identified four genes most correlated with overall survival in the training set (n=76). (**D**, **G**) Kaplan–Meier curves of overall survival based on the IPS in the training set and validation set (n=76). (**E**, **H**) ROC curve analysis of the IPS. (**F**, **I**) Risk scores distribution, survival status of each patient, and heatmaps of prognostic four-gene signature in the training set (n=76) and validation set (n=76).

LASSO Cox regression analysis was used to construct an IPS for the training set (Figure 4A–C). Risk scores were calculated for each sample (risk score= CXCL5*0.077+IGHV3-11*0.317+TNFSF14*0.296+LGR6*0.054). Patients in the training set were divided into a low-risk group and a high-risk group based on the optimal cut-off value (0.6979717) calculated through the “survminer” R package. The outcomes of patients with a high-risk score were worse than those with a low-risk score, as suggested by Kaplan–Meier analysis (Figure 4D). The Receiver Operating Characteristic (ROC) curve analysis of the IPS in the training set indicated a promising prognostic ability for OS (Figure 4E). Figure 4F shows the results of risk score distribution and gene expression patterns in the training set.

### Validation of the immune prognostic signature

To verify the prognostic value of the IPS, the same formula was applied to the validation set. Patients in the validation set were divided into high- and low-risk by the cut-off value obtained from the corresponding cohort. Similar to the results of the outcomes in the training set, the patients with a high-risk score tended to have worse OS (Figure 4G). The ROC analysis displayed high sensitivity and specificity of the IPS (Figure 4H). The Area Under the Curve (AUC) values for predicting 1- and 2-year patient survival were 0.633 and 0.695, respectively, demonstrating the high predictive value of the IPS. Figure 4I shows the results of risk score distribution and gene expression patterns in the validation set. The prognostic value of the IPS in the total set containing the training, validation, and WHO grade III gliomas sets is shown in Supplementary Figure 2.

### Establishment of an IPS-based nomogram model

**Figure 5:**
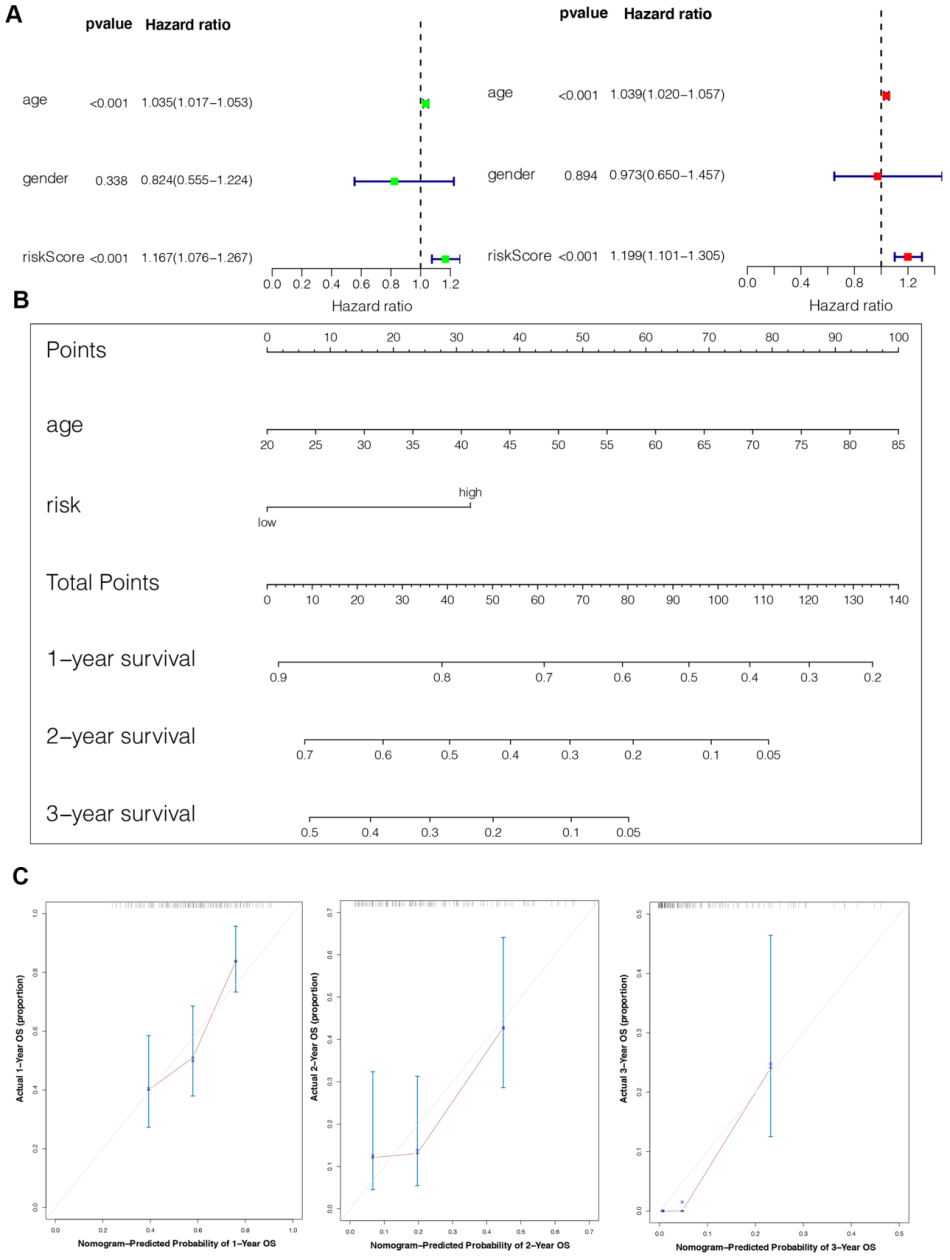
**Construction of the nomogram model.** (**A**) Univariate and multivariate Cox analyses indicating that the IPS is significantly associated with OS. (**B**) Nomogram model for predicting the probability of 1-, 2-, and 3-year OS in GBMs. (**C**) Calibration plots of the nomogram for predicting the probability of OS at 1, 2, and 3 years.

The univariate Cox analysis revealed that the IPS was significantly associated with OS (Hazard ratio: 1.167, 95% confidence interval: 1.076−1.267, *P* < 0.001). The multivariate Cox analysis showed that the IPS could serve as an independent prognostic factor (Hazard ratio: 1.199, 95% confidence interval: 1.101−1.305, *P* < 0.001) (Figure 5A). Finally, we established an IPS-based nomogram model (Figure 5B). The C-index (0.674) showed the specific discrimination ability of the nomogram model. Calibration plots of observed vs. predicted probabilities of 1-, 2-, and 3-year OS demonstrated favorable concordance (Figure 5C).

## DISCUSSION

Foxp3, a unique marker of natural regulatory T cells (nTregs) and adaptive/induced regulatory T cells (a/iTregs) [19], is a protein involved in immune system responses [20]. Foxp3^+^ Tregs are considered to constitute an essential part of the immunosuppressive microenvironment of gliomas [21]. However, the mechanism regulating the interaction of Foxp3 with the immune microenvironment is yet to be established.

Of all the GBMs, we found that mesenchymal glioblastomas have higher expression of Foxp3 than proneural and classical tumors (Supplementary Figure 3A). Kaffes et al. also demonstrated that mesenchymal glioblastomas are characterized by an increased immune cell presence [22]. No significant difference was observed between different IDH status in GBM (Supplementary Figure 3B).

In the present study, although we found that immune cells, functions, and pathways were enriched in Foxp3_H, the patients still had a worse prognosis. In contrast, He et al. found that triple-negative breast cancer patients with high immune status had a better prognosis [15]. In the analysis of HLA expression, we found that most HLA genes were expressed higher in Foxp3_H. Previous studies have shown that HLA expression was correlated with tumor grade and histological type [23]. Fan et al. found that glioma patients with low HLA-DR expression were more likely to benefit from immunotherapy [24]. Hence, immunotherapy might be more effective in patients of Foxp3_L.

The glioma immunosuppressive microenvironment might be responsible for these observations. We found that the expression of BTLA, CD27, ICOS, LAG-3, TIM-3, and IDO1 was higher in Foxp3_H (Figure 2E). This result suggested that, although immune activity was higher in Foxp3_H, immune cells were generally depleted and did not function optimally. Previous studies have also shown tumor-associated lymphocyte infiltration in glioblastoma with the lymphocyte generally having a poor function and severe exhaustion [25]. Therefore, immune checkpoint inhibitors could be a more effective treatment option for patients with high immune checkpoint gene expression.

To confirm the effect of Foxp3 in immune regulation, we compared Foxp3_H and Foxp3_L using differential expression analysis. A total of 294 genes were identified as the DEGs, including 91 differentially expressed immune genes among them. From the GO and KEGG analyses, the DEIGs were significantly enriched in complement activation, classical pathway, phagocytosis, recognition, T cell co-stimulation (GO terms), and cytokine-cytokine receptor interaction (KEGG). Furthermore, we developed and validated a Foxp3-related IPS which was related to prognosis. The four-gene IPS was also an independent prognostic factor by univariate and multivariate Cox analyses. Moreover, a predicting nomogram was developed based on the IPS to predict the survival of patients with GBM.

Four genes were identified as hub genes in our IPS by LASSO Cox regression: *CXCL5*, *IGHV3-11*, *TNFSF14*, and *LGR*6. *CXCL5* (C-X-C motif chemokine ligand 5) encodes the protein CXCL5, a small cytokine belonging to the CXC chemokine family that is produced upon the stimulation of cells with the inflammatory cytokines IL-1 or TNF-α [26]. CXCL5 is not only known to regulate neutrophil homeostasis [27] but is also related to cancer cell migration/invasion and tumor angiogenesis [28]. In previous research, CXCL5 was shown to be up-regulated in glioma tissues compared with normal brain tissues and could promote the proliferation and migration of glioma cells by activating the ERK, JNK, p38, and MAPK signaling pathways [29]. In glioma cells, CD133, a cancer stem cell marker, raises the expression of IL-1β and its downstream chemokines, including CCL3, CXCL3, and CXCL5 [30]. Foxp3^+^Tregs could exert an immune function by controlling the CXCL5-IL-17 inflammatory axis [31]. And CXCL5 caused a 2.5-fold increase in the frequency of Foxp3^+^Tregs in CD4^+^T cells [32]. *IGHV3-11* (immunoglobulin heavy variable 3-11) was one of the VH3 immunoglobulin gene family. The molecular function of *IGHV3-11* is related to antigen binding and immunoglobulin receptor binding. Previous study had demonstrated that immunoglobulin could expand Foxp3^+^Tregs [33]. *TNFSF14* (TNF superfamily member 14) encodes a member of the tumor necrosis factor (TNF) ligand family. This protein has been shown to induce the expansion of T cells and induce apoptosis of different tumor cells [34]. Treps et al. fused a CGKRK peptide with TNFSF14 and injected it intravenously into murine orthotopic GBM models. The tumor vasculature appeared to have normal features after treatment. Additionally, the authors observed more endothelial venules and T cell infiltration in solid tumors [35]. In a recent study, researchers determined that the expression of TNFSF14 negatively correlates with tumor mutation burden (TMB) in GBM and that TNFSF14 is a significant prognostic factor for poor OS [36]. TNFSF14 can promote the proliferation of Foxp3^+^Tregs [37], and bind HVEM which are expressed on Foxp3^+^Tregs to mediate the suppressive functions [38]. *LGR6* (leucine-rich repeat containing G protein-coupled receptor 6) encodes a member of the leucine-rich repeat-containing subgroup of the G protein-coupled 7-transmembrane protein superfamily. LGR6 binds to RSPO ligands to activate the Wnt/β-catenin signaling pathway to promote cancer progression [39]. And Wnt/β-catenin signaling could modulate the TCF-1-dependent inhibition of Foxp3 transcriptional activity to limit immunosuppressive activity [40].

Interestingly, immune checkpoint genes, BTLA, CD27, ICOS, LAG-3, TIM-3, IDO1, and PD-L1 were expressed higher in the high-risk group (Supplementary Figure 4). This indicated that the high-risk patients identified by the Foxp3-related IPS might be having a better effect on immune checkpoint inhibitors.

For now, some predictive models of glioblastoma have been constructed. Tang et al. identified a model of a specific gene module. The AUC values were 0.61-0.67 and 0.71-0.88 for predicting 12-months and 36 months patient’s survival, respectively [41]. A relatively authentic immune-related predictive model constructed by Liang et al. had the AUC value of 0.869 [42]. In our present study, we found Foxp3 expression to be associated with prognosis and developed a Foxp3-related IPS with the AUC values for predicting 1- and 2-year patient survival of 0.633 and 0.695, respectively in the validation set. Our result provided a novel vision to study glioblastoma and we will verify the accuracy of the Foxp3-related IPS by our data in further research works.

Currently, the research on the microenvironment of gliomas attracting considerable attention from the scientific community. This study provided new insights into the GBM immune microenvironment from different perspectives. Nevertheless, there were some limitations. This study was retrospective hence there is a need for further prospective studies to confirm the value of our four genes functionally and mechanistically.

In summary, our study has identified the mechanism of Foxp3 expression on prognosis from the perspective of immunology. We have constructed a Foxp3-related IPS that can classify patients into different immune risk groups. Moreover, we have developed a nomogram to quantitatively predict a patient’s survival based on this IPS. These findings are beneficial for GBM patients for the development of individualized treatment plans and improvement of prognosis.

## MATERIALS AND METHODS

### Data collection and immunohistochemical staining

The clinical and prognostic significance of Foxp3 expression was assessed in a prognosis cohort using immunohistochemical (IHC) staining of tissue microarray (TMA) slides. Formalin-fixed paraffin-embedded blocks of 72 specimens (49 WHO grade III gliomas and 23 WHO grade IV glioblastomas) were collected from the pathologic archive of the Sun Yat-sen University Cancer Center (SYSUCC) from 1^st^ January 2003 to 1^st^ June 2006. Clinicopathological and follow-up data were retrieved from the medical records. The staging and grading evaluation followed the World Health Organization (WHO) 2016 classification. All the procedures in the current study were approved by the ethics committee of Sun Yat-sen University Cancer Centre. Written informed consent was obtained from all the patients.

Immunohistochemical staining for Foxp3 (1:1000, ab10901) was conducted on 4-μm-thick TMA sections according to the manufacturer's instructions. Immunoreactivity in >0% of cells was defined as positive Foxp3 IHC staining. Two pathologists read the Foxp3 IHC slides.

### Gene expression datasets and data processing

RNA-seq data from 152 GBM samples were obtained from the TCGA website (https://portal.gdc.cancer.gov/). The survival data were available for all the patients, and their survival time was at least 30 days. Patients were divided into two groups, 76 Foxp3_H patients and 76 Foxp3_L patients, based on their median Foxp3 expression value. The RNA transcriptome profiling was then performed using a log2-based transformation of FPKM values.

### Immunogenomic analysis

Enrichment levels of the 29-immune signature in each GBM sample were quantified using the single-sample gene-set enrichment analysis (ssGSEA) score [16, 17]. This was followed by the evaluation of the immune cell infiltration level (immune score), stromal content (stromal score), and tumor purity for each GBM sample by Estimation of STromal and Immune cells in MAlignant Tumours using Expression data (ESTIMATE) [43]. Expression levels of human leukocyte antigen (HLA) genes and immune checkpoint genes were then compared between Foxp3_H and Foxp3_L using analysis of variance (ANOVA) test.

### Survival analysis

The OS of GBM patients was compared between Foxp3_H and Foxp3_L. The log-rank test was used to calculate the significance of survival time differences using a threshold of *P* < 0.05. Kaplan–Meier curves were plotted to show the survival time differences.

### Differential expression analysis

Differential expression analysis between Foxp3_H and Foxp3_L was processed and executed onRstudio using the Wilcoxon Rank Sum and Signed Rank Tests [44]. Genes with log2 |fold change| ≥1 and False Discovery Rate (FDR) <0.05 were selected as differentially expressed genes (DEGs). DEIGs were identified by the ImmPort database (https://www.immport.org/) [18].

### Functional enrichment analysis

To analyze gene ontology and signaling pathway enrichment, DEIGs were uploaded to an online tool, Metascape, a website for gene annotation, visualization, and attributes (https://metascape.org/) [45–48].

### Construction and validation of the immune prognostic signature (IPS)

For the construction of the immune prognostic signature (IPS), the TCGA GBM dataset was randomly divided into two sets: training and validation. DEIGs in the training set were placed into LASSO Cox regression for analysis using the “glmnet” R package to establish IPSs [49–51]. An IPS was created by weighting the Cox regression coefficients to estimate a risk score for each patient. Patients were categorized as low-risk or high-risk according to the optimal cut-off values acquired by the “survminer” R package. Receiver operating characteristic (ROC) curves were generated to evaluate the sensitivity and specificity of IPS using the “survivalROC” R package [52]. The area under the curve values were calculated for the ROC curves. Subsequently, the prognostic prediction power of this IPS was further validated using the validation set.

### Development of the nomogram

The univariate and multivariate Cox analyses were used to evaluate the independent prognostic ability of the IPS. The “rms” package was used to develop an innovative nomogram based on the results of the Cox analyses. Calibration plots of observed vs. predicted probabilities of 1-, 2-, and 3-year OS were generated to determine the accuracy. The discrimination of the model was determined using the concordance index (C-index). Bootstraps were calculated to correct the C-index [53].

## Supplementary Material

Supplementary Figures

Supplementary Tables
