# Supplementary material for: A novel Foxp3-related immune prognostic signature for glioblastoma multiforme based on immunogenomic profiling
Source: Aging (Albany NY). 2021 Jan 10;13(3):3501–17. doi: 10.18632/aging.202282 (PMC7906197; doi:10.18632/aging.202282)
Supplement: Supplementary Figures [file aging-13-202282-s001.pdf]

SUPPLEMENTARY FIGURES

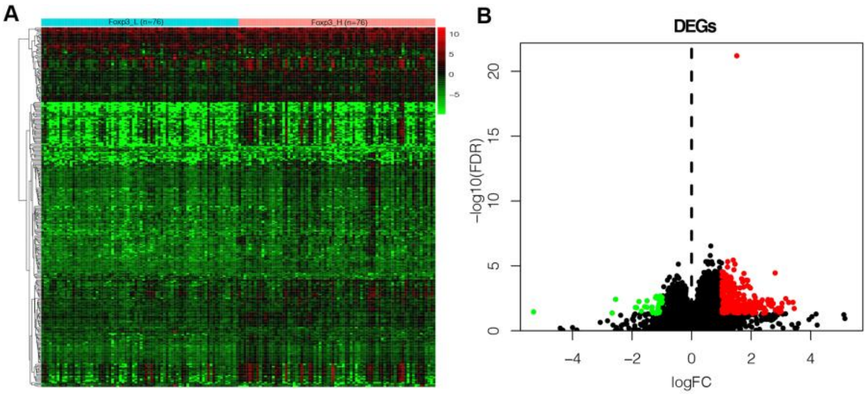

Supplementary Figure 1. Identification of Foxp3-associated differentially expressed genes. (**A**) Heatmap of genes differentially expressed between Foxp3_H and Foxp3_L. (**B**) Volcano plot of 294 genes differentially expressed between Foxp3_H and Foxp3_L.

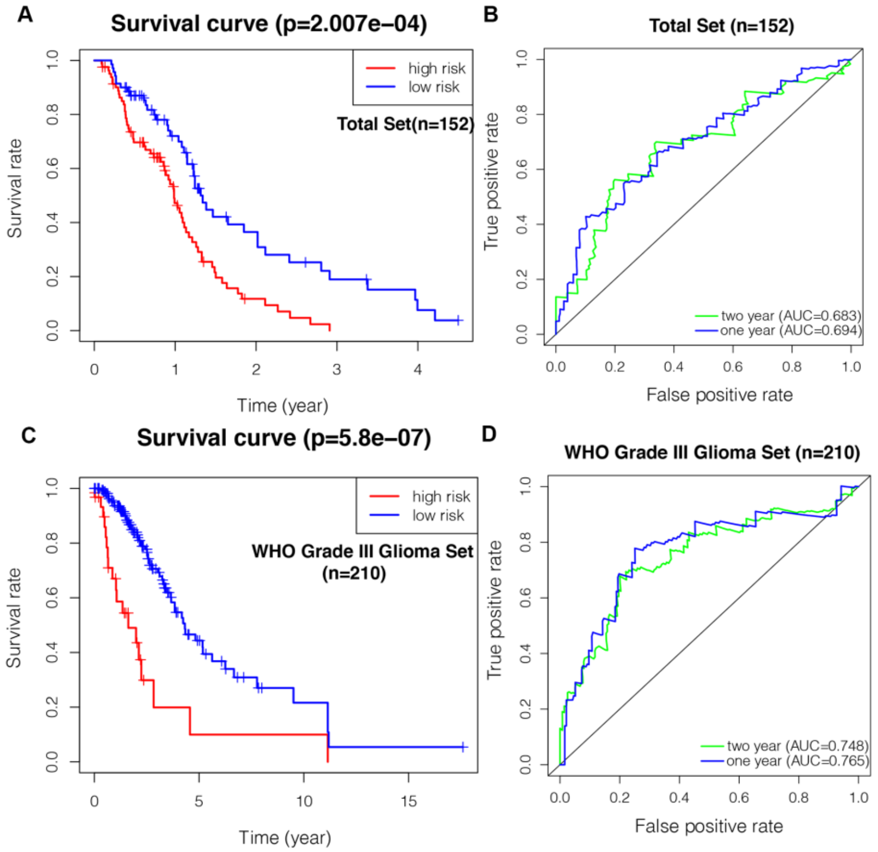

Supplementary Figure 2. The prognostic value of the IPS in the total set, containing the training set, the validation set, and WHO grade III gliomas set. (**A**) Kaplan–Meier curves of overall survival based on the IPS in the total set (n=152). (**B**) ROC curve analysis of the IPS in the total set (n=152). (**C**) Kaplan–Meier curves of overall survival based on the IPS in the WHO grade III gliomas set (n=210). (**D**) ROC curve analysis of the IPS in the WHO grade III gliomas set (n=210).

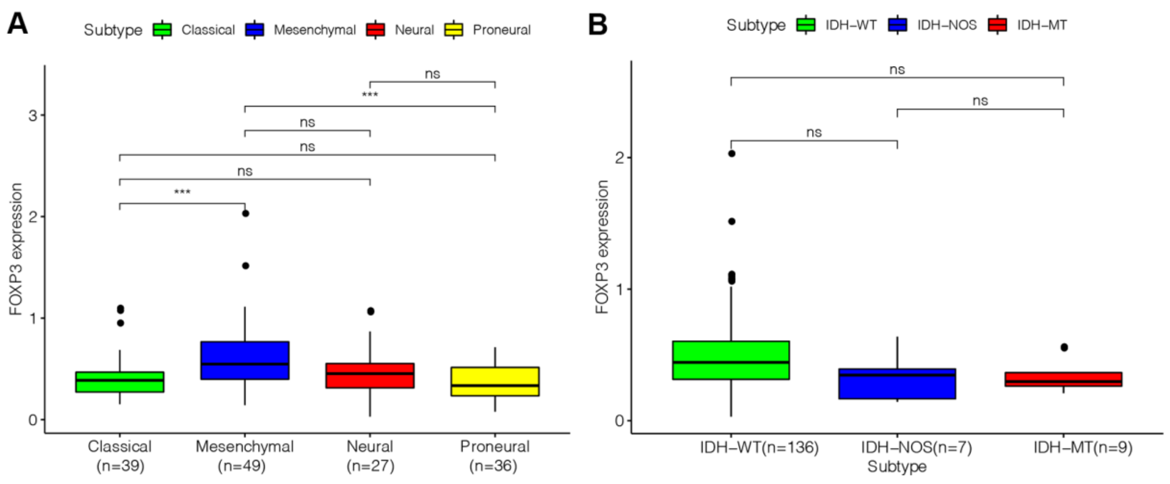

Supplementary Figure 3. FOXP3 expression in GBM with different subtypes and IDH status. (**A**) FOXP3 expression in GBM with different subtypes. (**B**) FOXP3 expression in GBM with IDH status. *$P < 0.05$, **$P < 0.01$, ***$P < 0.001$.

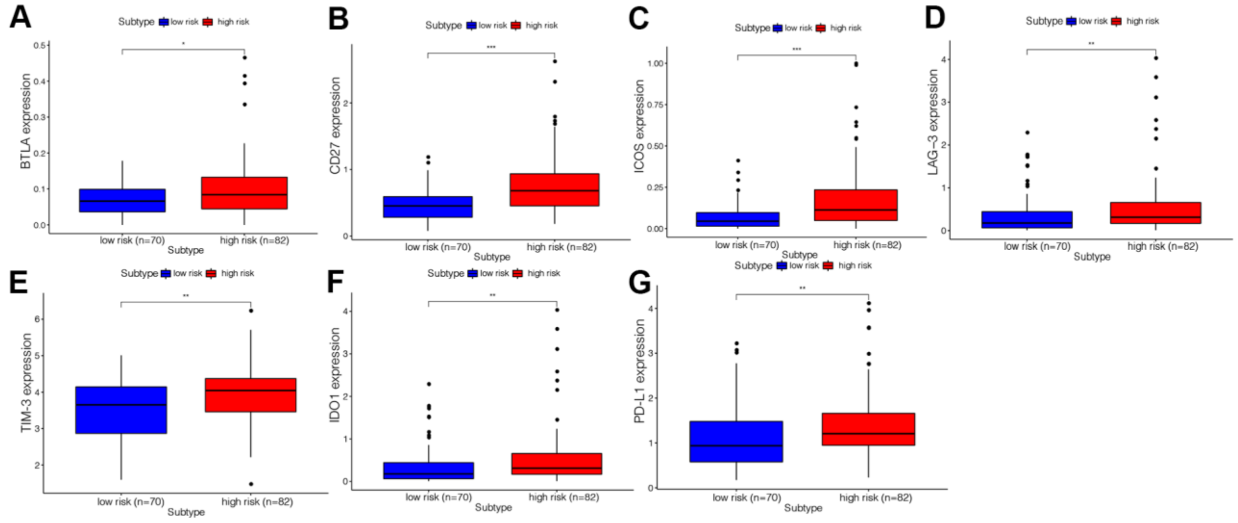

Supplementary Figure 4. Comparison of immune checkpoint gene expression levels between high risk and low risk using ANOVA test. *$P < 0.05$, **$P < 0.01$, ***$P < 0.001$.
